# Biosimilar monoclonal antibodies in China: A patent review

**DOI:** 10.1080/21655979.2022.2090206

**Published:** 2022-06-26

**Authors:** Jia-Wei Liu, Yu-Huan Yang, Nan Wu, Ji-Fu Wei

**Affiliations:** a Department of Pharmacy, Jiangsu Cancer Hospital & Jiangsu Institute of Cancer Research & the Affiliated Cancer Hospital of Nanjing Medical University, Nanjing, Jiangsu, China; b Department of Clinical Pharmacy, School of Basic Medicine and Clinical Pharmacy, China Pharmaceutical University, Nanjing, Jiangsu, China; c Department of Clinical Pharmacy, School of Pharmacy, Nanjing Medical University, Nanjing, Jiangsu, China

**Keywords:** Biosimilar, monoclonal antibody, Chinese patent, cancer, autoimmune disease

## Abstract

Biosimilars play an important role in reducing the burden on patients and increasing the market competition. Biosimilar monoclonal antibodies are currently one of the hotspots of research and development in China with policies support. With the continuous improvement of policies, the enthusiasm for the research and development of biosimilars has increased year by year. The policy requirements in different periods have different degrees of impact on the patent applications of pharmaceutical companies. This review introduces the biosimilar monoclonal antibodies market status and approval process in China, analyzes the patents in this field, and helps pharmaceutical companies protect their intellectual property rights.

## Graphical Abstract

**Figure:**
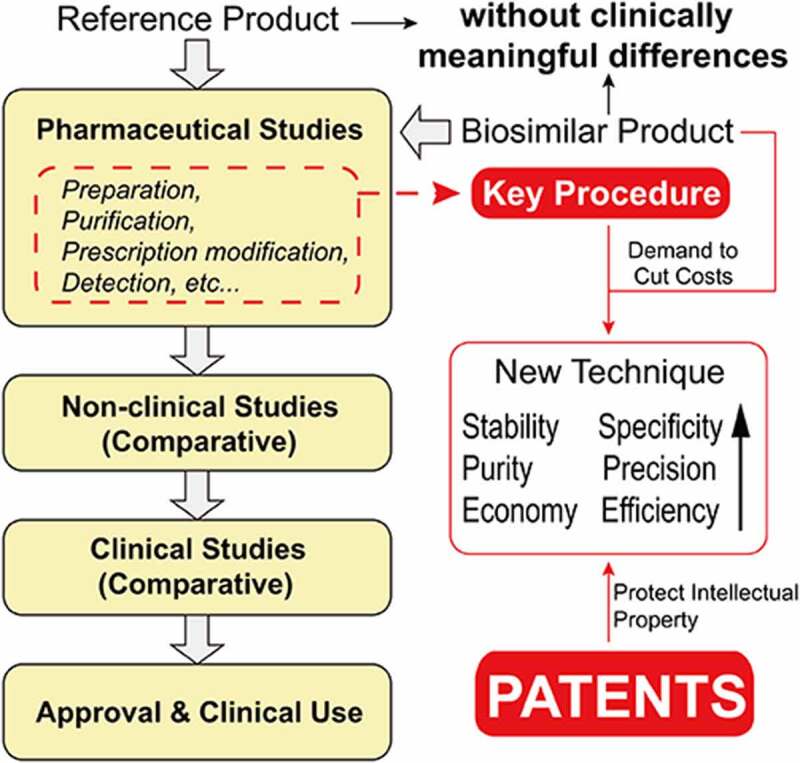

## Highlights

- A summary of the patents related to biosimilar mAbs in China and their advantages.
- An overview of Chinese biosimilar monoclonal antibody market and development.
- Patents that improve the prescription of preparations account for the majority.

## Introduction

Monoclonal antibodies (mAb), first commercialized in 1986, have already developed into the vital therapeutics of diseases, especially tumor and autoimmune diseases [1,2]. Up to 2021, there are over 40 mAbs launched in China, most of which are imported. Compared with chemotherapeutic drugs, mAbs provide more efficacy, more specificity, but cost more. The extravagant medicinal cost becomes a burden to patients and society, which impedes the development of the mAbs market in China. In 2018, the world’s best sell drug was adalimumab (Humira®), while no mAbs ranked in the top 10 in China[3]. Meanwhile, there are huge gaps in technique and equipment between home and abroad. The market of mAbs in China still has room for growth.

Because of rising competition from biosimilars, the price of mAbs decreases and access increases. For instance, in China, the price of trastuzumab (Herceptin®) in 2016 was 24,500 and then decreased to 7600 after involved in the medical insurance list. In 2020, Zercepac®, a biosimilar of Herceptin®, was listed at 1688 in China, helping patients receive efficacious and economical treatment. The 13th Five-Year Plan also pointed out the significance of developing biosimilars and regarded biosimilars as an important part of novel biomedical system. Different from generic drugs, biosimilars cannot be the same as original drugs due to their large molecular mass, complex structure, and undisclosed manufacturing process[4].

**Figure 1.**
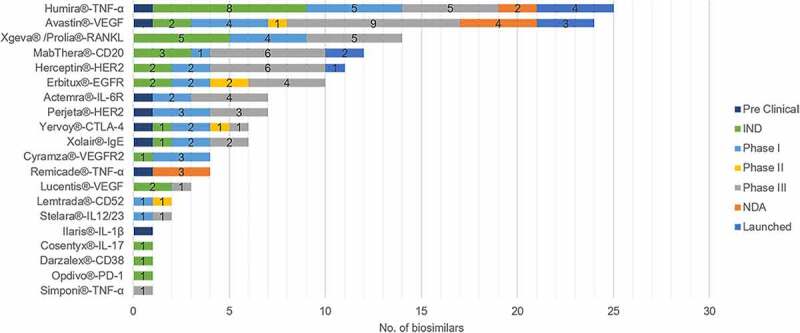
Numbers of the research and development of different biosimilars in China. Adalimumab and bevacizumab are the hotspots in the Chinese biosimilar market, while mAbs with new targets show less competitions.

Since the first biosimilar, Rituximab injection copied by Henlius, was launched in 2019, 142 biosimilar mAbs involved 16 targets have been researched and developed in China, and ten of them have been launched (Figure 1). China has over 60 pharmaceutical companies in this field. Representative companies include Henlius, Hisun, Mabpharm, Qilu Pharmaceutical, Hualanbio, Biotech, Chiatai Tianqing, and Innoventbio. Table 1 shows the outline of biosimilar mAbs of these companies and their trial progress.

**Table 1.** Representative companies and the R&D progress of their biosimilar products.

Companies	Original drug	Name	Indication	Trial number	Phase	R&D status
Henlius	Rituximab	HLX01	DLBCL	CTR20150583	3	Listed in 2019
	Adalimumab	HLX03	PS	CTR20171123	3	Listed in 2020
	Trastuzumab	HLX02	BC	CTR20160526	3	Listed in 2020
	Bevacizumab	HLX04	CRC	CTR20171503	3	Recruited
	Cetuximab	JZB28/9	HNSCC	CTR20210716	1	Not yet recruiting
	Pertuzumab	HLX11	BC	CTR20200618	1	Not yet recruiting
	Denosumab	HLX14	PMO	CTR20201905	1	Recruiting
	Ramucirumab	HLX12	GC	CTR20190389	1	Recruiting
	Daratumumab	HLX15				IND
	Ipilimumab	HLX13				IND
Hisun	Adalimumab	HS016	AS	CTR20160398	3	Listed in 2019
	Bevacizumab	MIL60	Non-squamous NSCLC	CTR20170658	3	NDA
	Infliximab	HS626	RA	CTR20180351	3	NDA
	Rituximab	HS006	DLBCL	CTR20180855	3	Recruiting
	Trastuzumab	HS022	BC	CTR20180362	3	Recruiting
	Pertuzumab	HS627	BC	CTR20200737	3	Not yet recruiting
	Tocilizumab	HS628	RA	CTR20201263	3	Not yet recruiting
	Omalizumab	HS632	Asthma	CTR20200763	1	Not yet recruiting
	Denosumab	HS629	Bone metastasis	CTR20180258	1	Not yet recruiting
Mabpharm	Infliximab	CMAB008	RA	CTR20170934	3	NDA
	Omalizumab	CMAB007	Asthma	CTR20170959	3	Recruited
	Cetuximab	CMAB009	CRC	CTR20170701	3	Recruiting
	Denosumab	CMAB807	PMO	CTR20202319	3	Recruiting
	Tocilizumab	CMAB806	RA	CTR20190739	3	Recruiting
	Trastuzumab	CMAB809	BC	CTR20190897	1	Completed
	Adalimumab	CMAB815				IND
	Pertuzumab	CMAB810				Pre-clinical
	Canakinumab	CMAB816				Pre-clinical
Qilu	Bevacizumab	QL1101	NSCLC	CTR20161024	3	Listed in 2019
	Trastuzumab	QL1701	BC	CTR20192189	3	Recruiting
	Cetuximab	QL1203	CRC	CTR20191318	3	Recruiting
	Pertuzumab	QL1209	BC	CTR20201073	3	Recruiting
	Denosumab	QL1206	PMO Bone metastasis	CTR20190726 CTR20191911	3 3	Recruited Recruiting
	Ranibizumab	QL1205	AMD	CTR20191290	3	Not yet recruiting
	Ramucirumab					IND
Hualan Bio	Adalimumab	HL01	RA/AS/PS	CTR20200016	3	Recruiting
	Rituximab	HL03/WBP263	DLBCL	CTR20190424	3	Recruiting
	Trastuzumab	HL02	BC	CTR20190665	3	Recruiting
	Bevacizumab	HL04	Non-squamous NSCLC	CTR20181297	3	Recruiting
	Cetuximab	HL07/WBP297	CRC	CTR20190662	1/2	Not yet recruiting
	Ipilimumab	HL06/WBP299	Melanoma	CTR20190661	1/2	Recruiting
	Denosumab	HL05	Bone metastasis	CTR20191886	1	Not yet recruiting
Biotech	Adalimumab	BAT1406	AS	CTR20160565	3	Listed in 2019
	Bevacizumab	BAT1706	Non-squamous NSCLC	CTR20170799	3	NDA
	Golimumab	BAT2506	PsA	CTR20210172	3	Not yet recruiting
	Ustekinumab	BAT2206	PS/CD/UC PS	CTR20200461 NCT04728360	1 3	Not yet recruiting Not yet recruiting
	Tocilizumab	BAT1806	RA	CTR20190174	3	Recruited
	Secukinumab	BAT2306				IND
Chiatai Tianqing	Adalimumab	TQ-Z2301	AS	CTR20181863	3	NDA
	Rituximab	TQB2303	DLBCL	CTR20182377	3	Recruiting
	Trastuzumab	TQB211	BC	CTR20181909	3	Recruiting
	Bevacizumab	TQB2302	Non-squamous NSCLC	CTR20180857	3	Recruiting
	Pertuzumab	TQB2440	BC	CTR20201685	3	Recruiting
	Ramucirumab		GC/NSCLC/CRC	CTR20191906	1	Completed
Innoventbio	Adalimumab	IBI303	AS	CTR20160628	3	Listed in 2020
	Rituximab	IBI301	DLBCL	CTR20160493	3	Listed in 2020
	Bevacizumab	IBI305	Non-squamous NSCLC	CTR20160848	3	Listed in 2020
	Ipilimumab	IBI310	HCC	CTR20210080	3	Recruiting
	Denosumab	IBI307				IND

Henlius and Innoventbio have the most biosimilar products on the market; nevertheless, Innoventbio has fewer biosimilars than other companies.

In the United State of America and the European Union, the development of biosimilar products is equally rapid. Filgrastim-sndz (Zarxio®) is the first biosimilar product approved by Food and Drug Administration (FDA) in March 2015. Up to April 2022, FDA approved 35 biosimilar products and 28.6% (10/35) were approved in 2019. [5]. Somatropin (Omnitrope®) is the first biosimilar product in the world and was approved by European Medicines Agency (EMA) on April 12^th^, 2006. Since then, 68 biosimilar products were approved by EMA, and 17 were refused and withdrawn[6]. As for biosimilar mAbs, 18 and 32 biosimilar products were approved respectively by FDA and EMA. Both of these biosimilar mAbs showed similar effects and safety to their reference drugs. [7–14] The markets of the USA and EU are more competitive and energetic than that of China. Interestingly, Zercepac® (trastuzumab) from China was authorized by EMA in July 2020. [6]. The manufacturer of Zercepac® Henlius has reached partnerships with Eurofarma Laboratórios S.A., Accord Healthcare, Cipla, Mabxience, and other pharmaceutical companies to actively explore overseas markets[15].

The booming market of biosimilars requires powerful patent protections. Patent application is an important approach to protect their rights and interests. We searched and selected the patents from the web of China National Intellectual Property Administration (http://www.cnipa.gov.cn).

This review aims to introduce the outline of biosimilar approval process, summarize the patents related to biosimilar mAbs in China and their advantages, produce an overview of Chinese biosimilar mAb market, and help pharmaceutical companies protect their patents.

## Biosimilar development, review, and approval

**Figure 2.**
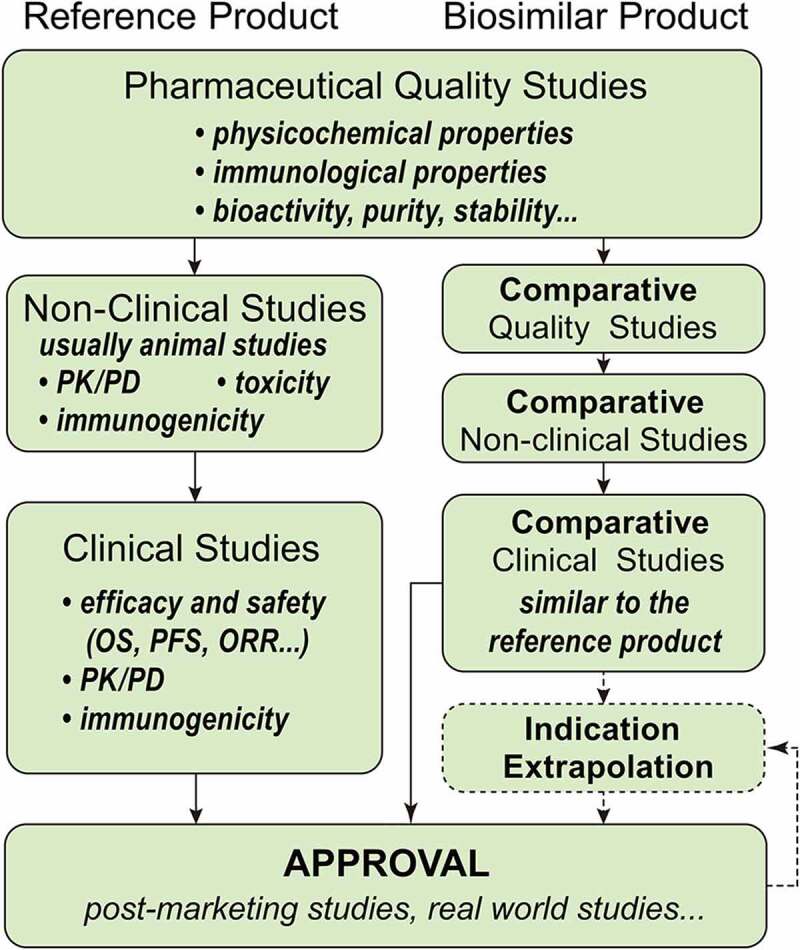
The developing process of the reference and biosimilar products. The elements of their processes are similar, both containing pharmaceutical studies, non-clinical studies (animal studies), and clinical studies. However, biosimilar products pay more attention on comparative studies and their indications can be extrapolated.

In 2015, the concept of biosimilar was first defined by National Medical Products Administration (NMPA) in China, with the announcement of Technical Guidelines for the Development and Evaluation of Biosimilars (Trial)[16]. Since then, the relevant systems have been continuously improved, forming a complete approval system for biosimilars. Figure 2 shows the development process of the reference and biosimilar product.

### Definitions and scope of application

According to the definition, the biosimilar is a therapeutic biologic that is similar in quality, safety and efficacy to the reference product, usually the original product, that has been approved for registration. The guideline emphasized that for modified products such as polyethylene glycol and antibody-coupled drug products, careful consideration should be given when developing biosimilars[16].

Same as FDA, the similarity of biosimilars means they have the same amino acid sequence, structure, purity, chemical identity, bioactivity, and other product characteristics. Some minor differences are acceptable, such as host cells, prescriptions, and acceptable within-product differences. The differences between the biosimilar and reference products should be no meaning in safety and effectiveness [16,17]. Different with FDA, the interchangeable product does not exist in the guideline. The patients need a prescription from prescribers written specifically for biosimilar to receive the biosimilar product[18].

### Development and review

The requirements of FDA and NMPA in biosimilar development are similar. The manufacturer of a proposed biosimilar should provide the comparative data evaluated from a systematic process consisting of pharmaceutical studies, non-clinical studies (animal studies), and clinical studies [16,19].

Pharmaceutical studies include physicochemical properties, bioactivity, purity (impurities), immunological properties, and stability, demonstrating that the biological product is highly similar to the reference product[20].

The design of non-clinical studies depends on the results of pharmaceutical studies, usually containing pharmacokinetics, pharmacodynamics, immunogenicity, and toxicity. Clinical studies also include assessing immunogenicity, pharmacokinetics, pharmacodynamics and comparative clinical studies in one or more of the indications for which the reference product is licensed[21].

### Indication extrapolation

Both FDA and NMPA agree that a biosimilar can be approved for an indication without direct studies of the biosimilar in that indication. However, the extrapolation is not automatic [19,20].

NMPA reckons that the indication extrapolation requires the following conditions to be met at the same time: 1) The completed comparison studies have used sensitive clinical trial models and no clinical differences have been detected; 2) The clinically relevant mechanisms and/or associated receptors of indications are the same; 3) The safety and immunogenicity of the biosimilar have been fully evaluated, and there are no special or additional safety issues for the proposed extrapolated indications[20].

FDA evaluates all of the biosimilar product data from studies mentioned above to assess whether the differences between the biosimilar and the reference product may affect the indications or populations not studied by the biosimilar manufacturer. If no such differences are detected, extrapolation of these indications is generally supported[19].

## Patent review of biosimilar monoclonal antibodies

**Figure 3.**
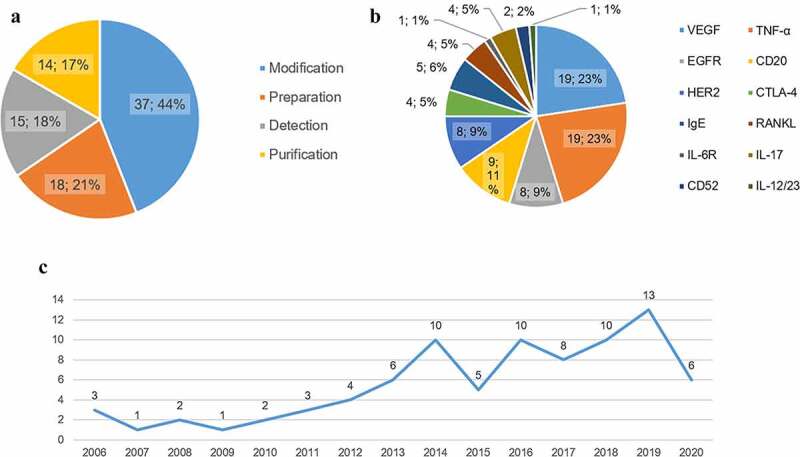
Outline of biosimilar patents in China. **a** The classification of biosimilar patents and the distribution. **b** Different targets of biosimilars and the patents of each target. **c** Trend analysis for Chinese patents on mAb biosimilars.

We found 84 patents for biosimilar mAbs, among 63 of which were in the protection or examination period. As shown in Figure 3, most of patents are about improvement of formulations. The patents authorized from 2015 to 2020 are approximate twice the number that before 2015 (52 versus 32). The main targets we focused on are tumor necrosis factor alpha (TNF-α), vascular endothelial-derived growth factor (VEGF), receptor activator of nuclear factor-kappa B ligand (RANKL), CD20, human epidermal growth factor receptor 2 (HER2), and epidermal growth factor receptor (EGFR). In addition, we summarized patents of other hot spots and novel targets.

### TNF-α-targeted antibodies

TNF-α is a cytokine produced by macrophages, lymphocytes, and natural killer cells and has effects on induction of systemic inflammation, regarded as a therapeutic target of autoimmune diseases [22–24]. Adalimumab, golimumab, and infliximab are available in China and have 25, 4, and 1 biosimilar respectively. Infliximab is a chimeric IgG1 antibody, approved for using in rheumatoid arthritis, Crohn’s disease, ankylosing spondylitis, psoriasis, and ulcerative colitis since listed in China in 2007. Adalimumab and golimumab have lower immunogenicity due to human IgG1 antibodies, also approved to be used in rheumatoid arthritis and ankylosing spondylitis. By May 2021, adalimumab biosimilars of Biotech, Hisun, Innoventbio, and Henlius have been listed. BAT1406 from Biotech was the first adalimumab biosimilar in China, bringing 200 million in sales. Listed in 2019, HS016 (Hisun) has similar safety, efficacy, and immunogenicity according to clinical trials (CTR20160450, CTR20160398, http://www.cde.org.cn) and is the first biosimilar approved for all indications of Humira® [25–27]. The clinical trial reports of IBI303 (Innoventbio, CTR20160219, CTR20160628, CTR20160687, http://www.cde.org.cn)[28] and HLX03 (Henlius, CTR20160930, CTR20171123, http://www.cde.org.cn)[29] supported the clinical development of two biosimilars.

There are 19 patents related to anti-TNF-α mAb biosimilar, including 9 (47.4%) patents about modification of prescriptions, 5 (26.3%) of preparation technique, 4 (21.0%) of purification, and 1 (5.3%) about detection method (Table 2). Lin *et al*. changed the prescription to enhance the stability of the antibody and allow intravenous injection. However, the concentration of BAT1406 cannot be over 80 mg/mL[30]. In 2019, Wu *et al*. improved the prescription in CN201310093009.7, the patent mentioned above, adding acetic acid, histidine-HCl, arginine-HCl, methionine, and Tween 80 to increase the upper limit of concentration by 50%[31].

**Table 2.** The patents of anti-TNF-α antibody biosimilar.

Patent	Title	Legal status
CN201210563488.X	Aqueous drug preparation of anti-TNF (tumor necrosis factor)-alpha human monoclonal antibody for strengthening stability	Licensing
CN201310093009.7	Human antibody preparation for treating TNF (tumor necrosis factor)-alpha related diseases	Licensing
CN201310611288.1	Stable anti-TNF-alpha antibody preparation and uses thereof	Licensing
CN201310693338.5	Adalimumab-containing pharmaceutical composition	Licensing
CN201410066863.9	Preparation method of recombinant adalimumab Fab fragment in escherichia coli	Deemed withdrawal
CN201510004710.6	Method for preparing novel recombinant anti-TNF alpha chimeric monoclonal antibody and application thereof	Substantive examination
CN201610156287.6	Method for purifying adalimumab by aid of cation exchange chromatography	Licensing
CN201610158762.3	Anti-TNF-alpha monoclonal antibody chromatographic method	Licensing
CN201610307094.6	A method for preparing a recombinant adalimumab Fab fragment in an insect cell expression system	Substantive examination
CN201710224783.5	Anti-freezing dry infliximab preparation	Substantive examination
CN201710224525.7	Infliximab composition containing histidine buffer system	Substantive examination
CN201710981104.9	Combined medium for expressing adalimumab	Licensing
CN201711146780.0	Method for purifying recombinant anti-TNF-alpha completely-human monoclonal antibody	Substantive examination
CN201711144785.X	Preparation method of recombinant anti-TNF-alpha completely humanized monoclonal antibody	Substantive examination
CN201811641361.9	Anti-tumor necrosis factor-alpha antibody injection preparation	Application publication
CN201910038495.X	Method for detecting ethanol residue in recombinant hominine anti-TNF-a project cleaning validation sample	Substantive examination
CN201910585391.0	Human antibody preparation for targeted therapy of TNF-alpha related diseases	Licensing
CN201911005798.8	Production method of anti-TNF-alpha monoclonal antibody	Application publication
CN202011188635.0	Method of removing recombinant expression antibody aggregates and degradation products	Licensing

The innovation in industrial manufacture process also needs to protect. Leng *et al*. invented a combined medium for expressing adalimumab to increase quantity and quality[32]. Xie *et al*. disclosed a production procedure of anti-TNF-α mAb improving purity and recovery rate[33]. Zhang *et al*. enhanced the specificity of total organic carbon (TOC) detection by adopting gas chromatographic (GC) to detect ethanol residue[34].

### VEGF-targeted antibodies

Researchers have confirmed the vital role of VEGF in cancers and blinding eye diseases[35]. VEGF is a family including VEGF-A (generally referred to as VEGF), VEGF-B, VEGF-C, VEGF-D, VEGF-E, and placental growth factor (PIGF), which regulates angiogenesis and lymphangiogenesis. VEGF receptors (VEGFR1, VEGFR2, and VEGFR3) were discovered in 1992 and VEGFR2 was the main receptor for VEGF[35].

Overexpression of VEGF in tumors correlates with majority, metastasis, recurrence, and prognosis. VEGF can also promote immune evasion of tumors by stimulating the proliferation of myeloid-derived suppressor cells and regulatory T cells[36]. As for eye diseases, neovascularization driven by VEGF can cause bleeding, retinal detachment, and fibrovascular proliferation, which turns to blindness[37]. The role of VEGF in the choriocapillaris is crucial for retinal vascular diseases.

Several mAbs targeted the VEGF-VEGFR signaling pathway have been launched. Bevacizumab, a VEGF-targeted humanized IgG1 antibody, was approved for the treatment of colorectal cancer (CRC), non-small cell lung carcinoma (NSCLC), and glioblastoma in China. Since listed in 2010, the price of Avastin® has decreased by 61.4%. In China, the antibody sequence patent of bevacizumab expired in 2018. Qilu Pharmaceutical’s bevacizumab was approved for marketing in 2019, becoming the first domestically produced bevacizumab biosimilar. Biosimilars of Innoventbio and Luye Pharma has been approved for listing, which significantly reduces patients’ medical cost. All biosimilars showed comparative efficacy, safety, and quality [38–42].

Ranibizumab is a humanized IgG1 Fab that is obtained from the same parental mouse antibody as bevacizumab, approved for age-related macular degeneration (AMD). Ramucirumab is a human IgG1 antibody targeted on VEGFR2 and not available in China. Biosimilars of ranibizumab and ramucirumab are still in research and development.

As shown in Table 3, there are 19 patents about VEGF-targeted biosimilar mAbs. 9 (47.4%) patents relate to prescription innovation, while patents about preparation, purification, and detection are 4 (21.0%), 3 (15.8%), and 3 (15.8%) respectively.

**Table 3.** The patents of anti-VEGF antibody biosimilar.

Patent	Title	Legal status
CN201110274419.2	Simple and convenient chemical industrial technology for prokaryotic expression and purification of humanized anti-vascular endothelial growth factor monoclonal recombinant antibody	Deemed withdrawal
CN201210185573.7	Method for purifying and preparing anti-VEGF antibody fragment	Licensing
CN201210579417.9	Preparation method of long-acting sustained-release microspheres containing bevacizumab	Deemed withdrawal
CN201310337426.1	Bevacizumab eye drop and preparation method thereof	Licensing
CN201410093781.3	Hypodermic high-density anti-VEGF antibody formulation	Licensing
CN201410198778.8	High-stability humanized antibody preparation for treating VEGF related diseases	Substantive examination
CN201410487742.1	Pharmaceutical composition of humanized antibody for vascular endothelial growth factor	Substantive examination
CN201410625267.X	Biological activity detection method for VEGF targeted therapy drugs	Deemed withdrawal
CN201410757524.5	Stable anti-VEGF antibody preparation and application thereof	Licensing
CN201510583416.5	Stable protein preparation	Deemed withdrawal
CN201610244018.5	Stable anti-VEGF (vascular endothelial growth factor) antibody preparation and application thereof	Rejection
CN201610817744.1	Purification method of anti-VEGF (Vascular Endothelial Growth Facto) type monoclonal antibody	Substantive examination
CN201611216530.5	Stable bevacizumab preparation	Deemed withdrawal
CN201710716867.0	Method for preparing highly-pure ranibizumab inclusion body	Deemed withdrawal
CN201810364192.2	High-producing strain with capability of efficient secretion expression of anti-VEGF-Fab antibody fragment, and construction method thereof	Substantive examination
CN201810355289.7	Method for efficient expression of antibody Fab fragments	Substantive examination
CN201810998933.2	Monoclonal antibody for neutralizing bevacizumab and application thereof	Licensing
CN201811563705.9	Purification method of proteins	Application publication
CN201911417826.7	Biological activity analysis method of recombinant anti-VEGFR2 monoclonal antibody and application thereof	Substantive examination

The patents of prescription focus on the stability of mAbs. Wu *et al*. changed buffer solution and stabilizer into a phosphate buffer solution and trehalose to enhance the stability of the antibody[43]. Wang *et al*. provided a buffer system containing 1.0–5.0 mg/ml of sodium acetate trihydrate, reducing the physical and chemical degradation reaction rates of mAbs[44]. Cheng *et al*. adopted a combination buffer system of a sodium phosphate buffer agent and a second buffer agent, and adopted one or two of mannitol or sodium chloride as an osmotic pressure regulator to significantly reduce polymers and degraded materials[45].

In addition, Liu *et al*. invented the two-step purification of cation exchange-hydrophobic chromatography with a purity of more than 95%[46]. Liu *et al*. offered a biological activity detection method meeting the requirements on the specificity, accuracy, precision, and other verifications[47]. To enhance the expression of ranibizumab, Li *et al*. invented a high-producing strain, deleting 11 nonessential regions in an *E. coli* DH1 genome[48].

### RANKL-targeted antibodies

Receptor activator of nuclear factor-kappa B (RANK) and its ligand (RANKL) are part of the TNF superfamily, most strongly expressed in bone. The functions of RANKL signaling include remodeling bone, immunity regulation, cell growth and differentiation, and the development of other organs [49,50]. Tumor metastasis affects the prognosis of patients, which is a hotspot in oncology. Tan *et al*. revealed that tumor-infiltrating CD4^+^CD25^+^FoxP3^+^T cells are a major source of RANKL production and stimulate metastatic progression, which explains more aggressive behavior in advanced breast cancers[51]. Osteoclasts are the main functional cells for bone resorption and play an important role in bone development, growth, repair, and reconstruction. RANKL regulates the differentiation and activation of osteoclasts. The imbalance of osteoclasts and osteoblasts may cause osteoporosis, which means RANKL can be a drug target for bone diseases[52].

Denosumab, a human IgG2 antibody, is the first mAb targeted RANKL, approved for giant cell tumor of bone, postmenopausal women with osteoporosis at high risk for fracture, and multiple myeloma and bone metastasis from solid tumors in China. Several clinical trials (NCT00926380, NCT00089674, NCT00321620, NCT00089791, NCT00523341, https://clinicaltrials.gov) [53–57] support the efficacy of denosumab. The market of denosumab biosimilars is budding but competitive. Nine biosimilars of Chinese pharmaceutic companies are in clinical study and 5 biosimilars are in investigational new drug (IND).

The patents are not booming as the market. There are four patents of denosumab biosimilars. Mei *et al*. provided a novel purification method and a redox system composed of dithiothreitol (DTT) and sodium sulfite to prepare the denosumab biosimilar[58]. Three patents about biological activity detection showed advantages in specificity, accuracy, precision, and efficiency. Liu *et al*. disclosed an ameliorated tartrate resistant acid phosphatase assay[59]. Ye *et al*. and Ding *et al*. provided different luciferase assays, the former focused on the preparation of plasmid, while the latter focused on the tool cell selection [60,61].

### CD20-targeted antibodies

Rituximab was the first mAb approved for clinical use in oncology which is a chimeric IgG1 antibody targeted CD20, treating non-Hodgkin’s lymphoma (NHL) and chronic lymphocytic leukemia (CLL)[62]. CD20 is a B-cell surface marker regulating the development and differentiation of B cells into plasma cells. Most NHLs originate from mature B cells, presented a malignant monoclonal proliferation of lymphoid cells. Chemotherapy combined with rituximab is the typical treatment to kill malignant CD20-positive cells [62–64]. Chronic lymphocytic leukemia is another malignant tumor related to lymphoid cells, which is characterized by the clonal proliferation and accumulation of B-cells. Chemoimmunotherapy with fludarabine, cyclophosphamide, and rituximab (FCR) is the first-line treatment of CLL and shows great efficacy and safety in long-term follow-up [65,66].

The patent of rituximab (Mab Thera®) expired in 2015, which stimulated the market of rituximab biosimilars. In China, 2 biosimilars from Henlius and Innoventbio have been listed in 2019 and 2020 respectively with 12 biosimilars in total.

9 patents were found in this field (Table 4). Liu *et al*. improved the prescription of anti-CD20 antibody, adding hyaluronidase to help hypodermic injection, which is the only patent about prescription improvement[67]. Several scientists paid their attention on increasing production. Song *et al*. established a large-scale high-expression production technology for 300 L of eukaryocytes, wherein the anti-CD20 monoclonal antibody (consistent to rituximab) expression quantity is more than 1.2 g/L and the protein purification yield is improved to more than 60%[68]. Shen *et al*. established an efficient expression system whose yield of rituximab is 1.7–2.2 g/L, which laid a foundation for large-scale industrial production[69]. Zhang *et al*. improved the chromatographic performance by adding arginine, glycine, or mannitol in the ion-exchange chromatography process and adjusting electric conductivity with salt, having advantages in operation, cost, and safety[70]. Liu *et al*. disclosed a detection method for anti-CD20 mAb binding activities based on flow cytometry (FCM) with good specificity, precision, linearity and range, accuracy, and durability[71].

**Table 4.** The patents of anti-CD20 antibody biosimilar.

Patent	Title	Legal status
CN201110302889.5	Detection method for anti-CD20 monoclonal antibody binding activities	Licensing
CN201210356034.5	Anti-CD20 monoclonal antibody, preparation method, and application thereof	Lapsed
CN201310470054.X	Method for producing anti CD20 antibody	Licensing
CN201410052926.5	High-sensitivity anti-CD20 monoclonal antibody and applications thereof	Licensing
CN201410640266.2	ELISA method for quantitatively determining concentration of recombinant human-mouse chimeric anti-CD20 monoclonal antibody in human blood serum	Licensing
CN201710087562.8	High-expression and high-stability CHO cell line for producing Rituximab and constructing method thereof	Substantive examination
CN201810135774.3	Method for purifying anti-CD20 human-mouse chimeric monoclonal antibody	Substantive examination
CN201811542658.X	Antibody glycol-form modification formula, cell culture method, and application in industrial production	Application publication
CN201911157456.8	Medicine preparation containing anti-CD20 antibody as well as preparation method and application of medicine preparation	Application publication

### HER2-targeted antibodies

HER2 (also called receptor tyrosine-protein kinase ErbB-2) from ErbB family, is a product of proto-oncogene. The ErbB pathway is a complex biology signaling network that regulates the apoptosis, migration, growth, adhesion, and differentiation of cells [72–74]. The HER2 overexpression leads to the occurrence and invasion of tumors and can increase the risk of metastasis, which can be observed in 25–30% of breast and ovarian cancers[73]. Recent researches found HER2 overexpression in other solid tumors such as gastric cancer, biliary tract cancer, colorectal cancer, NSCLC, and bladder cancer[75].

Anti-HER2 therapy is a typical treatment for HER2-positive breast cancer, such as trastuzumab and pertuzumab (anti-HER2 humanized IgG1 antibodies)[76]. According to several clinical trials (NCT00567190, NCT00567190, NCT02131064, the drug combination of trastuzumab, pertuzumab, and chemotherapy showed efficacy in HER2-positive metastatic breast cancer, which benefits these patients [77–80]. However, the price of these mAbs hinders patients from effective treatment.

The availability of Zercepac® changed actuality. Produced by Henlius, Zercepac® is the first biosimilar of trastuzumab and has obtained all the indications that Herceptin® (trastuzumab) has been approved in China due to the pleasure results of clinical trials (NCT02581748, NCT03084237, ; CTR20160526, [81].,[82] Up to 2021, 18 biosimilars of trastuzumab and pertuzumab have entered the competition of anti-HER2 biosimilar.

There are 8 patents related to the biosimilars, 6 of which were valid. Wang *et al*. changed α,α-Dicarboxylic trehalose into sucrose or trehalose, reducing the cost of trastuzumab preparation[83]. Ma *et al*. provided a new pharmaceutical preparation of HLX11, the biosimilar of pertuzumab, containing sorbitol to control the budget[84]. Wu *et al*. replaced the L-histidine by a histidine-hydrochloride buffer in the prescription of pertuzumab [85] As for purification technology, Xu *et al*. improved and disclosed the purification method with simple steps, high recovery rate, and low potential virus risk[86]. In 2016, Li *et al*. modified the cation-exchange chromatography packing, increasing the productivity further[87]. Xie *et al*. simplified the procedure of purification and removed impurities effectively [88,89].

### EGFR-targeted antibodies

EGFR is a transmembrane protein of the ErbB family, similar to HER2. The EGFR pathway regulates cancer-cell proliferation, apoptosis blocking, invasion, and metastasis, and the mutation or overexpression of EGFR exists in different human cancers, which means EGFR is an ideal target for tumor therapy[89]. The activation of EGFR transduces the Ras/MAPK pathway, the most important pathway in EGFR mediation related to growth, survival, and differentiation of cells [90,91]. Cetuximab (Erbitux®), an anti-EGFR chimeric IgG1 antibody, was used to metastatic colon cancer and head and neck squamous cell carcinoma (HNSCC). Due to the treatment failure caused by Ras mutation, cetuximab is not suitable for Ras-mutated patients.

The patent of Erbitux® has expired in 2017; however, there are only 10 biosimilars in China and none of them complete their clinical trials. Kelun, Annpobio, and Mabpharm are pushing their phase 3 clinical trials (CTR20202451, CTR20192102, CTR20170701).

8 patents were selected from the database, while three patents were valid. He *et al*. donated an accurate and effective anti-EGFR monoclonal antibody biological activity detection method; the relative standard deviation using the method of the present invention was 4.2%, and the average recovery rate was 103.8%[92]. Qian disclosed an improved method that using CHO cells as host cells to prepare a new anti-EGFR with different glycoforms[93]. Yu *et al*. disclosed a screening culture method of cells capable of efficiently expressing an anti-EGFR mAb without fucosyl modification, which enhanced the antibody-dependent cell-mediated cytotoxicity (ADCC) effect [94,95].

### Other monoclonal antibodies and their biosimilar patents

The market of newborn mAbs in China shows less liveliness than that of mAbs mentioned in the previous article, which also explains fewer patents of their biosimilars. However, biosimilars and patents cannot completely reflect the market of mAbs due to the existence of innovative drugs.

CD38 and programmed cell death 1 (PD-1) are the targets that have no biosimilar patents. CD38 is a glycoprotein located on the membrane, which can catalyze the synthesis and degradation of cyclic adenosine diphosphate ribose and has high expression in multiple myeloma (MM) cells[95]. The current research on CD38 and tumors confirms that CD38 has important functions in promoting tumor cell growth and immune escape[96]. A human IgG1 antibody daratumumab is the only anti-CD38 mAb approved for MM and not launched in China. The IND of Henlius’s biosimilar HLX15 has been approved by NMPA.

PD-1 is a checkpoint that has been studied deeply in recent years. It is confirmed that PD-1 plays a vital role in balancing immunity and tolerance; however, high expression of PD-1 ligand (PD-L1) on tumor cells can lead to immune escape[97]. Nivolumab (Opdivo®) is a typical PD-1 mAb treating NSCLC, HNSCC, and gastric cancer (GC), having a biosimilar (LY01015) in IND. There are four innovative PD-1 mAbs in China, sintilimab (TYVYT®), camrelizumab (Airuika®), tislelizumab (Baizean®), and toripalimab (Tuoyi®), which enriches the market and provides more choices for patients.

Cytotoxic T Lymphocyte antigen 4 (CTLA-4), also called CD152, is the other checkpoint and expressed by regulatory T cells (Treg). In normal, CTLA-4 binds CD80/CD86 to avoid conventional T cells (Tcon) stimulation, which is of significance to prevent autoimmune diseases. In the tumor microenvironment, overexpression of CTLA-4 on Treg leads to the downregulation of immune responses [98,99]. Anti-CTLA-4 mAb can cut off the pathway and enhance the immune responses to tumor cells, such as human IgG1 antibody ipilimumab (Yervoy®). There are six biosimilars of ipilimumab in China and Innoventbio are undergoing their phase 3 clinical trial (CTR20210080, http://www.cde.org.cn). Four patents were found in the database. Scientists from Innoventbio, DongFang Biotech, and Shanghai Celgen disclosed their prescriptions, respectively [100–102].

Omalizumab is a humanized IgG1 anti-IgE mAb approved for asthma. IgE, produced by B cells in response to allergen, plays a vital role in the inflammatory of asthma. Omalizumab inhibits allergic reactions and downregulates the expression of IgE receptors to alleviate symptoms and prevent recurrence[103]. Six biosimilars and three patents with validity were found. These patents ameliorated the stability and quality of the anti-IgE mAbs [104–106].

CD52 is a membrane glycoprotein expressed on both B cells and T cells, but not expressed on CD34-positive lymphocytes. Alemtuzumab is a humanized IgG1 antibody against CD52, approved for CLL and multiple sclerosis (MS). However, Campath® (alemtuzumab for CLL) was not on the market in 2012 due to high toxicity. There are two biosimilars but no patent in legality.

Interleukins (ILs) are cytokines with low molecular weight produced by lymphocytes, macrophages, and monocytes, involved in the immune response and cell signaling [107–109]. A variety of ILs have been widely studied and developed as new therapeutic targets and most of the mAbs against these ILs are approved for autoimmune diseases, such as ustekinumab (anti-IL-12/23), tocilizumab (anti-IL-6), and secukinumab (anti-IL-17). Tocilizumab has more biosimilars in China than the other two mAbs, and there are 6 biosimilars in clinical trials (CTR20190174, CTR20190739, CTR20201263, CTR20190002, CTR20191204, CTR20192563, http://www.cde.org.cn). Canakinumab is a human IgG1 antibody against IL-1β approved for cryopyrin-associated periodic syndromes and other spontaneous inflammatory diseases. According to the company announcement, Mabpharm has commenced the pre-clinical research of canakinumab biosimilar. Five patents of anti-IL mAbs were found and four patents were valid. Lin *et al*. provided a novel prescription of BAT1806, the biosimilar of tocilizumab[110]. Ouyang *et al*. improved the technique of removing cysteinylated variant from secukinumab product[111].

## Discussion

The high price of most bioproductions, leading to high medical costs of related diseases, has affected the cost control of national health insurance and the sustainability of financial health expenditure. With the expiration of patents and intellectual property rights for the original drugs, more and more pharmaceutical companies are working on the development of low-cost biosimilars, which benefits patients and medical insurance payers from effective and relatively economical treatment. Biosimilars have emerged as a result.

In March 2015, NMPA in China announced the ‘Technical Guidelines for the Development and Evaluation of Biosimilars (Trial)’, which clarified the definition of biosimilars for the first time, proposed the basic principles for the development and evaluation of biosimilars, and put forward specific requirements for the contents of pharmacy, non-clinical and clinical research and evaluation of biosimilars. In July 2016, the Registration Management Measures (Amendment) further regulated the concept of biosimilars and tightened the approval criteria for biosimilar. Similar with the FDA and the EMA [112], biosimilars have not been given a simple approval but adopted the same approval as innovative bioproducts, which raised the requirement of pharmaceutical companies and promoted their development invisibly. In December 2017, the National Development and Reform Commission (NDRC) in China issued the Three-Year Action Plan to Enhance the Core Competitiveness of Manufacturing (2018–2020), encouraging the development and industrialization of bioproducts with high market potential, high clinical value, and expired patents, regarding the first biosimilar drug as a high-end drug, which helps technical cooperation and the import of advanced technology.

With the policies guiding and the needs of companies’ development, the overall innovation awareness of the biopharmaceutical industry in China has increased. Since the guide of biosimilar announced in 2015, the quantity of patents of biosimilar mAbs has gradually raised.

Patents that improve the prescription of preparations account for the majority. Usually, the excipients and buffer systems used in the original biologic products are of good quality and stable but high-priced. The manufacturers of biosimilars aspire to seek the same stable and lower-cost alternative excipients and substantiate that such changes do not generate clinical differences that affect the efficacy and safety.

Meanwhile, the transformation of new technologies in the field of biochemistry is also related to the booming growth of patents. When evaluating the quality characteristics of mAb products or formulating quality evaluation standards, a detecting technique with high sensitivity, selectivity, specificity is needed, which encourages manufacturers paying more attention on technology fundamentals and refinements. For instance, enzyme-linked immunosorbent assays (ELISA) are an important technique for pharmacokinetic studies of mAbs. However, due to the interference of endogenous proteins, anti-drug antibodies (ADAs), and soluble target ligands, the application of ELISA faces limitations[113]. Some of the patents about detection method provides new capture antibodies, new detecting systems, and combination methods to overcome these challenges.

With these improving techniques and scientific studies, the similarity of the marketed anti-tumor biosimilars with their reference drugs in terms of clinical efficacy, safety, and immunogenicity has been confirmed in the results of different registered clinical trials and clinical studies [25–27,29,38–41,81,82,114,115].

However, the protection of intellectual property should be multidimensional. Compared with the USA and EU, China could pay more attention to data exclusivity and market protection. According to current laws and regulations, data exclusivity and market protection are out of implementation. In the Measures for the Administration of Drug Registration (2007 Version) released by NMPA, 6-year-data exclusivity for new chemical drugs and 5-year-market protection for new drugs were valid but are invalid in the latest version[116]. Optimistically, a draft for comments was released by NMPA on May 9, 2022, emphasizing the importance of data exclusivity and market protection[117]. It will be a concrete implementation of encouraging pharmaceutical innovation and a huge improvement for intellectual property protection. Due to the development of biotechnology and bioproducts markets, the market size of biosimilars in China will gradually grow with the improvement of the national guidelines.

## Conclusion

In this article, we reviewed the patents of biosimilar mAbs in China. A total of 263 patents were found, among which 84 patents are related to biosimilar mAbs. In total, 63 (75.0%) of these patents are in the protection period. The main targets of biosimilar mAb patents are VEGF, TNF-α, CD20, EGFR, and HER2.

## Abbreviation

eADA: anti-drug antibody; ADCC: antibody-dependent cell-mediated cytotoxicity; AMD: age-related macular degeneration; AS: ankylosing spondylitis; BC: breast cancer; CD: Crohn’s disease; CLL: chronic lymphocytic leukemia; CRC: colorectal cancer; CTLA-4: Cytotoxic T Lymphocyte antigen 4; DLBCL: diffuse large B-cell lymphoma; DTT: dithiothreitol; EGFR: epidermal growth factor receptor; ELISA: enzyme-linked immunosorbent assay; EMA: European Medicines Agency; FCM: flow cytometry; FCR: fludarabine, cyclophosphamide, and rituximab; FDA: Food and Drug Administration; GC: gastric cancer; HCC: hepatocellular carcinoma; HER2: human epidermal growth factor receptor 2; HNSCC: head and neck squamous cell carcinoma; IL: interleukin; IND: investigational new drug; mAb: monoclonal antibodies; MM: multiple myeloma; MS: multiple sclerosis; NDA: new drug application; NDRC: National Development and Reform Commission; NHL: non-Hodgkin’s lymphoma; NMPA: National Medical Products Administration; NSCLC: non-small cell lung carcinoma; PD-1: programmed cell death 1; PLGF: placental growth factor; PMO: post-menopausal osteoporosis; PS: psoriasis; PsA: psoriatic arthritis; RA: rheumatoid arthritis; RANK: receptor activator of nuclear factor-kappa B; Tcon: conventional T cell; TNF-α: tumor necrosis factor alpha; TOC: total organic carbon; Treg: regulatory T cell; UC: ulcerative colitis; VEGF: vascular endothelial-derived growth factor
